# Gut Microbiota and Obesity: A Role for Probiotics

**DOI:** 10.3390/nu11112690

**Published:** 2019-11-07

**Authors:** Ludovico Abenavoli, Emidio Scarpellini, Carmela Colica, Luigi Boccuto, Bahare Salehi, Javad Sharifi-Rad, Vincenzo Aiello, Barbara Romano, Antonino De Lorenzo, Angelo A. Izzo, Raffaele Capasso

**Affiliations:** 1 Department of Health Sciences, University Magna Graecia, Campus “Salvatore Venuta”, 88100 Catanzaro, Italy; 2 Clinical Nutrition Unit, and Internal Medicine Unit, “Madonna del Soccorso” General Hospital, Via Luciano Manara 7, 63074 San Benedetto del Tronto (AP), Italy; scarpidio@gmail.com; 3 CNR, IBFM UOS, Università degli Studi “Magna Graecia” di Catanzaro, Campus “Salvatore Venuta”, 88100 Catanzaro, Italy; carmela.colica@cnr.it; 4 Greenwood Genetic Center, Greenwood, SC 29646, USA; lboccuto@ggc.org; 5 Clemson University School of Health Research, Clemson, SC 29634, USA; 6 Student Research Committee, School of Medicine, Bam University of Medical Sciences, Bam 44340847, Iran; bahar.salehi007@gmail.com; 7 Zabol Medicinal Plants Research Center, Zabol University of Medical Sciences, Zabol 61615585, Iran; javad.sharifirad@gmail.com; 8 Department of Medical and Surgical Sciences, University of Magna Græcia, Viale Europa, Germaneto, 88100 Catanzaro, Italy; vincenzoaiello91@gmail.com; 9 Department of Pharmacy, School of Medicine and Surgery, University of Naples Federico II, Via Domenico Montesano 49, 80131 Naples, Italy; barbara.romano@unina.it (B.R.); aaizzo@unina.it (A.A.I.); 10 Division of Clinical Nutrition and Nutrigenomic, Department of Biomedicine and Prevention, University of Tor Vergata, Via Montpellier 1, 00133 Rome, Italy; delorenzo@uniroma2.it; 11 Department of Agricultural Sciences, University of Naples Federico II, Via Università 100, 80055 Portici (NA), Italy

**Keywords:** metabolism, gut microbiota, dysbiosis, obesity

## Abstract

Nowadays, obesity is one of the most prevalent human health problems. Research from the last 30 years has clarified the role of the imbalance between energy intake and expenditure, unhealthy lifestyle, and genetic variability in the development of obesity. More recently, the composition and metabolic functions of gut microbiota have been proposed as being able to affect obesity development. Here, we will report the current knowledge on the definition, composition, and functions of intestinal microbiota. We have performed an extensive review of the literature, searching for the following keywords: metabolism, gut microbiota, dysbiosis, obesity. There is evidence for the association between gut bacteria and obesity both in infancy and in adults. There are several genetic, metabolic, and inflammatory pathophysiological mechanisms involved in the interplay between gut microbes and obesity. Microbial changes in the human gut can be considered a factor involved in obesity development in humans. The modulation of the bacterial strains in the digestive tract can help to reshape the metabolic profile in the human obese host as suggested by several data from animal and human studies. Thus, a deep revision of the evidence pertaining to the use probiotics, prebiotics, and antibiotics in obese patients is conceivable

## 1. Introduction

Changes in dietary habits and the increased availability of high-caloric foods have made overweightness and obesity some of the most serious health issues of our era. In 2016, the World Health Organization (WHO) estimated that 39% of individuals older than 18 were overweight, and the worldwide prevalence of obesity almost tripled between 1975 and 2016. It has been reported that nearly 2.8 million deaths annually are a consequence of overweight and obesity-associated conditions: blood hypertension, dyslipidemia, and insulin resistance lead to an increased risk of coronary heart disease, ischemic stroke, type 2 diabetes mellitus, as well as cancer development [1]. Obesity is caused by an imbalance between energy intake and expenditure: increasing the intake of fattening food and other lifestyle changes pushed its prevalence to epidemic proportions. On the other hand, several works have proven a significant genetic role in determining the obesity risk [2,3,4]. On top of the genetic factors clearly contributing to determining body weight and fat mass, the drastic boost in obesity prevalence has also suggested a prominent contribution in the development and maintenance of obesity caused by environmental elements.

In recent years, changes in bacterial strains, hosted in the human intestine, were proposed to have a causative role in obesity [3,5,6]. Intriguingly, the microbiome is a fingerprint of both the environment and human heritable genetic material [7]. In fact, it has been proposed that the genetic pool of the microbiota represents an extension of the nuclear and mitochondrial genomes, leading to the definition of the meta-genome to describe such extension [8].

The present work aims to provide a synthetic overview of the state of the art in terms of development, distribution and composition of the gut microbiota. Another aim of the review is the association between intestinal microbiota and obesity, focusing on different subgroup populations. Finally, we will highlight some shreds of evidence sustaining the hypothesis that gut microbes may contribute to the development of obesity and other associated chronic metabolic conditions.

### Literature Search Methodology

A systematic literature research was conducted using a number of relevant keywords (MeSH: metabolism, gut microbiota, dysbiosis, obesity, probiotics).

In particular, we considered review manuscripts, randomized-clinical trials (RCTs), and case-series reports, both in animals and in humans. 

We looked also for preliminary evidence from the databases of main national and international gastroenterology, nutrition, endocrinology meetings (e.g., Digestive Disease Week, United European Gastroenterology Week, etc.).

## 2. Historical and Current Perspectives

The complex interaction involving the diet, intestinal microbiota, and human host has been investigated for over a century. Acceptance of the germ theory of disease development led to an original classification of a number of human disorders as caused by microbes, including conditions that were eventually going to be reconsidered as non-infectious. The initial proponent of such theories was the immunologist Elie Metchnikoff, considered by many as the father of probiotics. In his 1907 article, ‘‘Essais optimistes’’, Metchnikoff proposed a causative link between microbes and aging mechanisms and suggested a central role in senescence progression for compounds resulting from microbial intestinal putrefaction [9]. Furthermore, he firstly noted the beneficial effect of consuming fermented food on human health. Therefore, he hypothesized that fermented foods could avoid intestinal proteins putrefaction and thus senility.

Over the past century, several studies have demonstrated the influence of gut microbiota on the pathophysiology of many extra-intestinal conditions. More specifically, the exhaustive description of human microbiota’s relationship with health and disease has become the major challenge of research in the twenty-first century [10]. In recent years, the number of annual publications on this topic has almost quadrupled, as compared to 2005, when Eckburg et al. published the first large-scale gut metagenomic study that, starting from genetic fragments, allowed the reconstruction of entire genomic germ profiles [11].

Gut microbiota is the most complex ecosystem in nature since it harbors large bacterial populations in the intestine and colon, with around 1011–1012 microorganisms/gram of the intestinal content and mostly are anaerobes (95% of the total organisms) [12]. The first studies on the composition of intestinal microbiota were based on microscopic observation and culture-based methods, and showed as predominant cultivable species *Bacteroides* spp., *Eubacterium* spp., *Bifidobacterium* spp., *Peptostreptoccocus* spp., *Fusobacterium* spp., *Ruminococcus* spp., *Clostridium* spp. and *Lactobacillus* spp. [13]. Subsequently, Gill et al. obtained the first metagenomic sequencing of the distal gut microbiome in two subjects, showing microbial genomic and genetic diversity and identifying some of the distinctive features of this subpopulation of microbiome [14].

To date, genetic tests have led to the generation of large new datasets on gut microbiota, including information on the composition and functional properties of greater numbers of microbial strains. In this frame, US National Institutes of Health (NIH), founded the Human Microbiome Project (HMP) Consortium. HMP follows into the footsteps of the Human Genome Project, being constituted by multiple projects that bring together scientific experts worldwide to explore microbial communities to characterize the composition of the normal microbiome and the relationship with human organism [15]. Characterizing the microbial genes has led to the description of a human microbiome core [16]. It is established by a set of genes shared by microbes colonizing most habitats in humans. Interestingly, core genes present in a limited habitat and in a smaller set of humans can be modified by a combination of factors, such as host genotype, immune system physiology, disease state, lifestyle, diet, and also the presence of other microorganisms. This core microbiome is not present in shared big microbial populations but is involved in several essential metabolic functions for the bugs hosted in our intestine [17]. 

## 3. Gut Environment: Microbiota Evolutionary Development

The microbes detected in the human intestinal tract can be divided into three domains based on molecular phylogeny (i.e., 16S ribosomal ribonucleic acid [rRNA] sequence similarities and differences): eukarya, bacteria, and archaea. Eukarya includes organisms whose cells contain complex structures surrounded by membranes, primarily the nucleus. On the other hand, bacteria are the predominant strains of the gut microbiota (Table 1). About 90% of the fecal bacteria belong to two of the major phylogenetic lineages: *Firmicutes* and *Bacteroidetes*. However, the gastrointestinal tract of adult humans has been estimated to contain approximately 500–1000 distinct bacterial species [16]. In addition, *Methanobrevibacter smithii* is the dominant methanogenic archaeon species within the microbes in our digestive system [11]. 

More specifically, the subclass distribution of gut microbiota are composed by: Bacteroidetes (23%) that comprise the genus *Bacteroides*, Firmicutes (64%) that includes Bacilli, Clostridia and Mollicutes (the majority of microorganisms in this phylum are closely related to genus *Streptococcus* and *Clostridium*); Proteobacteria (8%), Gram-negative bacteria such as *Escherichia coli* and *Helicobacter pylori*; Fusobacteria, Verrucomicrobia and Actinobacteria (3%) that include species such as *Bifidobacterium* [18,19,20]. Over 20 genera of Bacteroidetes have been described, with *Bacteroidales* being the most studied one, in particular the genus *Bacteroides*. Firmicutes are Gram-positive bacteria, divided into three classes: *Clostridia, Bacilli,* and *Mollicutes* (Table 1).

Childhood is characterized by the microbial plasticity that resembles the physiologic process of progressive gut colonization by microbes over time. The colonization of the digestive apparatus begins at birth and is different from individual to individual [21]. This process recognizes three steps: from birth to weaning, from weaning to a normal diet assumption that is characteristic of adulthood, and elderly. More particularly, at birth, the human gut is essentially free from bacteria, but, immediately after delivery, the intestine begins to be populated by a series of microorganisms—this process is influenced by exogenous and endogenous factors (e.g., mother’s vaginal and fecal microbiota, environment, skin bacterial flora) [22]. During the first 12–24 h of extra-uterine life, gut colonists are especially facultative anaerobic bacteria such as *Escherichia coli*, *Enterococci* and *Streptococci* [22]. Subsequently, from the second to the third day, these bacteria generate an anaerobic environment promoting the growth of obligate anaerobes (*Lactobacilli* and mainly *Bifidobacteria*), perhaps through reduction of the redox environment potential (low oxygen concentration). Within two weeks, this bacterial population expands from 108 to 1010 per gram of feces and establishes itself as species *Bacteroides* and *Clostridia* [23]. A crucial determinant of gut microbiota development is the infant feeding. Several studies have shown different qualitative compositions of the bacterial flora in the breastfed subjects compared to the artificially fed ones. In breastfed infants, *Bifidobacterium* prevails (60%–90% of the fecal flora) vs. less than 1% of lactic-acid bacteria. In addition, there is a decrease in pH and inhibition of putrefactive flora growth with advantage for fermentative one development. This microbial switch improves intestinal digestive and absorptive functions of nutrients, in particular vitamins, with a consensual stimulation of immune system, namely gastrointestinal associated immune system (GALT), that reduces the risk of contracting allergies [24]. After the first six months of life, the weaning period begins with an enlarged diet composition and the introduction of the solid foods that leads to a further differentiation of microorganisms present in adults [25]. More specifically, these bugs belong to Firmicutes and Bacteroidetes (26). In the first year of life, levels of *Escherichia coli* and *Enterococci* range between 106 and 108 CFU/g of feces—there is a reduction in *Clostridia* and an increase in anaerobic flora, that undergoes a gradual diversification [24,25]. Interestingly, the initial colonization of the intestinal tract by microbes is important for defining the bacterial flora of the adult age. In fact, once the adult microbiota is constituted remains stable, with the exception of possible variations, following several factors such as a change in eating habits or the onset of diseases [26]. In adolescent children, a significantly higher representation of genera *Bifidobacterium* and *Clostridium* has been reported, as compared to adult levels [25,26]. A decline in the microbial abundance and species diversity, has been reported in the elderly, with lower levels of bifidobacteria and higher levels of Enterobacteriaceae [27].

## 4. Gut Microbiota Distribution and Its Relationship with Obesity

Differences in composition have been noticed in the microbial populations along the gastrointestinal tract [28]. These differences add a horizontal stratification, with the presence of diverse microbial communities in the intestinal lumen, in the layer of mucus of the intestinal crypts and directly adherent to the epithelial cells. In quantitative terms, esophagus and stomach carry the lowest bacterial load and the predominant cultivable bacteria are facultative anaerobes that derive from the oral cavity (e.g., *Streptococci* and *Lactobacilli)*. Bacterial load increases progressively along the intestinal tract as the redox potential drops. Moreover, the genus *Streptococcus* is the most represented among the microbiota of jejunum [28]. However, a significantly higher population of bacteria (108–109/g of feces) characterizes specifically the ileo-cecal area. In fact, the small intestine is enriched by the subgroup Bacillus bacteria (phylum Firmicutes, mainly Streptococcaceae, corresponding to 23% of the genomic sequences identified compared with 5% in the colon). In addition, up to 8% of genomic sequences belong to members of the phylum Actinobacteria and, in particular, to the subgroups Actinomycinaeae and Corynebacteriaceae. In the small intestine, a small percentage Bacteroidetes and Lachnospiraceae has been identified vs. their concentration in the colon [29]. The largest number of bacteria and the vastest microbial diversity (1011–1012/mL of luminal contents) in human gut have been observed in the distal section of the ileum and the colon. The greatest portion is composed by strictly anaerobic, often non-spore-forming, mainly Gram-positive (*Bacteroides* and *Clostridium*). There are also facultative anaerobes such as Lactobacillus, Enterococcus and Enterobacteriaceae [29,30]. This substantially higher concentration of bacteria is due to a slower motility characterized by anti-peristaltic contractions that allow retention of colonic content for long periods. In addition, the intestinal pH is buffered through the secretion of bicarbonate that makes the environment more favorable to the bacterial colonization [31].

The hypothesis that the intestinal microbiota can constitute to a relevant environmental factor in the pathogenesis of obesity has led to the investigation of gut microbial communities in overweight individuals. The first evidence indicating an association between obesity and intestinal microbes was produced by studies applying DNA sequencing methods on a large scale to allow the screening of the entire gut microbiome. The first link between gut microbial environment and obesity was hypothesized by Ley et al. that analyzed the gut microbiota of leptin-deficient mice at major phyla level [32]. Results from 16S rRNA gene sequencing in mouse models indicated as the two most abundant bacterial phyla were Firmicutes (60%–80%) and Bacteroidetes (20%–40%), and showed how mice homozygous for an aberrant leptin gene *ob/ob*, carried a different proportion of bacteria in the ceca compared to lean wild-type (*+/+*) or heterozygous (*ob/+*) mice. In particular, the *ob/ob* mice had a 50% decrease in the population of Bacteroidetes and a proportional increase in Firmicutes (*p* < 0.05). 

Similarly, Turnbaugh et al. published a study on mouse models using the newer shotgun metagenomic sequencing technique on cecal microbial DNA (*ob/ob*, *ob/+* and *+/+*) [33]. This study confirmed the increased ratio of Firmicutes vs. Bacteroidetes in obese mice, as compared to lean ones. Moreover, *ob/ob* mice had a higher proportion of Archaea within the cecal gut microbial communities. There was also a higher representation of genes involved in energy extraction from food in the obese host microbiota compared to lean host microbes. Works in another mammalian models noticed a lower abundance of Bacteroidetes associated with obesity [34,35]. Other works have associated mouse obesity with specific bacteria, in particular *Halomonas* and *Sphingomonas*, and the reduction in the *Bifidobacteria* number [36]. In order to assess if microbial communities can similarly affect weight gain or loss in humans, several studies have investigated various cohorts of obese and lean individuals, but the results have not always been consistent (Table 2).

### 4.1. Bacterial Species and Obesity

Numerous works have focused on the dynamics connecting changes in the levels of the major bacterial phyla Bacteroidetes and Firmicutes in relation to both obesity and weight loss. Ley et al. authored one of the first studies linking gut microbiota to obesity in humans: comparison of gut microbiota of lean and obese individuals revealed that obese subjects presented a reduced proportion of Bacteroidetes and higher levels of Firmicutes. More interestingly, after dietetic treatment the relative abundance of Bacteroidetes increased while that of *Firmicutes* decreased [32]. Armougom et al. assessed by real-time PCR the expression profiles of intestinal microbiota in different subjects and found a significantly reduced level of Bacteroidetes in obese versus lean (*p* < 0.01) or anorexic (*p* < 0.05) subjects, whereas Firmicutes concentration was similar in the three groups [37]. Subsequently, Million et al. aimed to assess the association of specific bacterial species, like *Lactobacillus* or *Bifidobacterium*, with obesity in a large case-control study that used part of patients of the cohorts investigated by Armougom et al. [37,38]. In particular, they analyzed the intestinal microbiota both at the phylum level, and within *Lactobacillus* and *Bifidobacterium* genera at the species level. Reduced levels of Bacteroidetes were detected in obese patients, although the difference with controls did not reach statistical significance (*p* = 0.25). Moreover, this study showed species-specific variations of Lactobacillus in obesity. *L. paracasei* resulted significantly associated with lean status, whereas *L. reuteri* and *L. gasseri* were significantly associated with obesity. Zuo et al. analyzed the composition of cultivable bacteria in obese and normal-weight subjects, and demonstrated that the amount of *Bacteroides*, the major Bacteroidetes genus and of *Clostridium perfringens*, was significantly lower in the obese group than in normal-weight one (*p* = 0.012 and *p* = 0.001, respectively) [39]. Other studies supported the findings of an opposite *Firmicutes/Bacteroidetes* ratio. Zhang et al. compared microbial community structures of nine individuals, three of each category of normal weight, morbidly obese and post-gastric-bypass surgery using 16S rDNA pyrosequencing technology [40]. The results indicated that H2-producing Prevotellaceae, within the class of Bacteroidetes, were significantly enriched in obese individuals compared to lean controls (*p* = 0.040). However, the difference in the relative abundance of Bacteroidetes was not significant between the two groups. Even if the results were generated from a cohort of small size, they provide the first hint on the link between energy homeostasis in humans and gut microbiota in obese subjects.

Another study investigating the difference in Bacteroidetes and Firmicutes proportion in obese subjects. reported that the median proportion of Bacteroidetes of the total sum of studied species was higher in overweight and obese vs. lean volunteers (*p* = 0.001 and *p* = 0.006, respectively) [41]. Furthermore, both overweight and obese subjects exhibited lower concentrations of *Ruminococcus flavefaciens* subgroup, part of the bacterial division of Firmicutes. Finally, a lower ratio of *Firmicutes/Bacteroidetes* was detected. However, it is notable that the methodology used in this study was questionable because the Bacteroidetes proportion was obtained by the sum of those of *Bacteroides* and *Prevotella* genera. Finally, the study reported reduction of *Clostridium leptum* group (*p* = 0.07) and of the *Bifidobacterium* genus (*p* = 0.02) in obese patients. 

On the other hand, a few studies did not find fine any correlation between gut microbiota composition and variations in body weight. However, they produced intriguing data that suggest hypotheses about the specific impact of several phyla or genera on obesity. Duncan et al. failed to detect a significant association between body mass index (BMI) or absolute weight loss and the relative concentration of populations of the major groups of human colonic bacteria, including Bacteroidetes, between obese and non-obese subjects [44]. Thus, they proposed that the evidence does not support a pivotal role for the proportion of Bacteroidetes and Firmicutes, at least at the phylum level, in predisposition to increased body weight. However, they found a significant diet-dependent reduction in *Eubacterium rectale/C. coccoides* levels, a group of butyrate-producing Firmicutes, in obese subjects on weight loss diet.

The composition of the intestinal microbiota of both African and Caucasian Americans was investigated by Mai et al. [45]. The proportions of Bacteroidetes and *Clostridia cluster XIV* (Firmicutes) have been analyzed by both qPCR and FISH techniques. The results did not show any association between altered microbiota composition and BMI, however they observed that individuals consuming high-fat diet had fewer *Clostridia*, while once consuming a fiber-reach diet there was an increase of lactic acid bacteria levels. Other studies have been performed in unique populations different for particular habits of life, or genetic and socio-economic status. Patil et al. reported a comparative analysis and quantification of the dominant gut microbiota of lean, normal, obese and surgically-treated obese individuals of Indian origin [54]. They detected no evident trend in the distribution of the predominant bacterial phyla, Bacteroidetes and Firmicutes by 16S rRNA sequencing. Indeed, the dominance of *Bacteroides* species at the genus level among obese individuals was further confirmed by means of RT-PCR that showed a positive correlation between *Bacteroides* and BMI (*p* = 0.002).

Combined analyses of different sequences databases from 33 gut microbiomes belonging to different nationalities led to the definition of three distinct clusters of enterotypes in the human microbiota on the basis of variation in the relative levels of *Bacteroides*, *Prevotella* and *Ruminococcus* [62]. Enterotype 1 is enriched in *Bacteroides* and co-occurring *Parabacteroides*, for which carbohydrates fermentation and proteins putrefaction represent the major energy sources. Enterotype 2 is enriched in *Prevotella* and *Desulfovibrio* that degrade mucin glycoprotein. Enterotypes 3 is the most frequent and is enriched in *Ruminococcus* and co-occurring *Akkermansia*, which degrades mucins. Enterotypes did not seem to differ in functional richness, and virtually none of several measured host properties as a nationality, gender or age, significantly correlated with any enterotype. Moreover, no correlation was found between BMI and Firmicutes/Bacteroidetes ratio [62]. 

Zupancic et al. enrolled old Amish subjects, due to their peculiar genetic pool, to study potential alterations of intestinal bacteria strains in obesity [55] and detected three networks of interacting bacteria in the human gut through 16S pyrosequencing analysis; these networks correlated with the three enterotypes of Arumugam study. The groups were respectively dominated by *Prevotella* genus group one, *Bacteroides* group two, and Firmicutes group three. Indeed, neither BMI nor any metabolic syndrome trait were associated with a particular gut community (*p* = 0.79).

Munukka et al. studied the peculiar population resembling 25% of obese people “metabolically healthy”, with normal lipid and glucose metabolism. The study aimed to evaluate eventual the differences in the intestinal microbiota of overweight/obese women with and/or without metabolic disorders [57]. Participants were divided into three groups: metabolic disease group (MDG), no-MD group (NMDG), and normal weight group (NWG), as control. There was a greater proportional amount of *Eubacterium rectale-Clostridium coccoides* group belonging to *Firmicutes* phylum, in MDG women, after adjusting for body weight, compared with NMDG and NWG. Also, MDG had a higher proportion of Gram-negative enteric bacteria than NW (*p* = 0.043), while the relative amounts of genus *Bifidobacterium*, *Atropium* cluster, *Bacteroides* group, and *F. prausnitzii* among three groups showed no significant differences. However, a larger concentration of *E. rectale-C. coccoides* positively correlated with body weight, BMI, FM, visceral fat area (all *p* < 0.01) and serum triglycerides (*p* < 0.05). On the other hand, *Bacteroides* inversely correlated with the same parameters (*p* < 0.05), but positively with HDL concentration (*p* < 0.01). The ratio of *E. rectale-C. coccoides* group and *Bacteroides* group, calculated by dividing the proportion of *E. rectale-C. coccoides* by the proportion of *Bacteroides*, was higher in the MDG. These findings showed how members of *E. rectale-C. coccoides* group are linked to obesity-related metabolic diseases, and not obesity alone. 

### 4.2. Archaea and Obesity

*Methanobrevibacter* is the main representative of Archaea in gut microbiota. Archaea are one of the first form of cellular life, together with Eubacteria. They do not present a single recognizable nucleus and have different cellular membrane compositions leading to their capability to be extremely resistant and impermeable to environmental factors [40]. Zhang et al. found a bigger abundance of *M. smithii* in obese individuals vs. lean controls [40]. Furthermore, Armougom et al. confirmed increased *M. smithii* levels in obese subjects vs. lean group [37]. Interestingly, re-evaluating these data as a means of log10 copies/mL *M. smithii* load was significantly higher in the obese group [57]. On the other hand, Schwiertz et al. found significantly lower levels of *M. smithii* in obese subjects compared to lean ones [41]. More recently, Million et al. found that *M. smithii* was less frequent and significantly less abundant in obese subjects [38,41]. 

From a mechanistic point of view, methanogenic Archea could indirectly promote caloric intake in the colon. The passage of hydrogen (H2) from H2-producing bacterium to H2-oxidizing methanogen can increase polysaccharides fermentation efficiency and removes fermentation intermediate products [38]. Furthermore, methanogenic Archaea could have a possible H2-producing bacterial partner namely *Prevotellaceae* (phylum *Bacteroidetes*). This partnership might lead to a greater efficiency of dietary polysaccharides fermentation, increasing their conversion into short-chain fatty acids with their excessive storage in obese subjects [40]. Indeed, a meta-analysis of the obesity-associated gut microbiota alteration at the genus level for *Methanobrevibacter* spp. revealed that obese subjects present less *Methanobrevibacter* than non-obese subjects [63]. This does not justify the above-mentioned hypotheses on the mechanisms responsible for excessive energy harvesting in obesity by methanogens. 

### 4.3. Bariatric Surgery and Gut Microbiota

Bariatric surgery has frequently been considered for anti-obesity treatment. To date, it is the sole option to provide substantial and persistent weight loss in severely obese patients [64]. Surgery can be performed according to several approaches such as reduction of the stomach size using an adjustable gastric banding, partial gastrectomy (sleeve gastrectomy, SG), creation of a small stomach pouch and, finally, resection and stomach re-routing into the small intestine (Roux-en-Y gastric bypass, RYGB). Utilization of these techniques implies substantial structural and functional modifications in the digestive system, particularly with the RYGB approach; for this reason, many attempts have been made to characterize changes occurring in the microbial composition of the distal portion of the intestine. A comparing study on three obese, three lean, and three post gastric bypass individuals revealed an increased proportion of *Gammaproteobacteria* (mostly *Enterobacteriacee*) and *Fusobacteriaceae* after surgery, accompanied by a proportional reduction of the levels of *Firmicutes* (namely *Clostridium* bacteria) and methanogens [40]. The authors hypothesized that the bypass of the upper small intestine can lead to the relocation of some typical bacteria of this tract (e.g., Enterobacteriaceae) to the large intestine; this modifies the gut microenvironment with consequent changes in food ingestion and digestion.

In a larger study, 30 obese subjects enrolled in a bariatric surgery follow-up program and 13 lean controls were assessed using a qPCR-based fecal microbiota analysis [51]. There was a standard increase in Firmicutes/Bacteroidetes ratio in obese patients before RYGB, and a subsequent decrease at three and six months postoperatively in agreement with patients’ weight loss. A noticeable correlation was reported between the levels of *F. prausnitzii*, *E. coli*, and *Bacteroides*/*Prevotella* and metabolic and inflammatory indexes. *F. prausnitzii* concentration was negatively correlated with serum concentrations of inflammatory circulating markers (hs-CRP, IL-6,). Interestingly, leptin levels fell after RYGB while *E. coli* concentration was significantly growing.

Patil et al. studied a group of surgically treated patients that underwent either sleeve-gastrectomy or adjustable gastric banding [54]. Treated–obese individuals exhibited significantly reduced *Bacteroides* spp. and Archaeal fecal counts, along with reduced fecal SCFA concentrations, studied by chromatographic analysis of fecal samples. Graessler et al. characterized intra-individual changes of gut microbiota before and three months after RYGB by metagenomic sequencing of fecal samples from diabetic type 2 obese patients (BMI > 40 kg/m^2^) [65]. The overall metagenomic RYGB-induced shift was characterized by a reduction of Firmicutes and Bacteroidetes and an increase of *Proteobacteria.* Twenty-two microbial species and 11 genera were significantly altered by RYGB: Proteobacterium *Enterobacter cancerogenus* was increased while Firmicutes *Faecalibacterium prausnitzii* and *Coprococcus comes* were decreased. These changes were associated with a significant improvement of weight control and of metabolic and inflammatory parameters. However, the authors observed that these shifts could have long-term effects on host health with a potential risk for bowel inflammation and colorectal cancer development. Li et al. examined rats gut microbiome after sham surgery or RYGB [66]. In accordance with Zhang et al., there was a significant decrease in *Firmicutes* to *Bacteroidetes* ratio, associated with a significant weight loss although in non-obese rats. Moreover, there was a significant increase in phylum Proteobacteria after RYGB. Subsequently, Liou et al. used a murine model of RYGB to assess whether changes in gut microbiota after gastric bypass surgery are consistent among humans, rats, and mice [67]. They showed that the microbial response to surgery depends only on the kind of surgery reshaping the gastrointestinal tract. Marked changes in gut microbial ecology were observed within one week after RYGB, with a pronounced increase in the abundance of the Verrucomicrobia (genus: *Akkermansia*) and *Gammaproteobacteria* (order Enterobacteriales, *Escherichia* spp.). These changes were similar to those observed in the fecal microbiota of human patients that had undergone RYGB. Very interestingly, these changes were not dependent on weight loss and/or caloric restriction, were detectable throughout the length of the gastrointestinal tract and were more significant in the distal gut. More interestingly, when transferring the microbiota of RYGB-operated mice to non-operated germ-free mice the latter experienced weight loss and decreased fat mass. On the other hand, recipients of microbiota induced by sham surgery did not show these changes. These findings support the link between changes in gut microbiota and reduced host weight and adiposity after RYGB surgery.

### 4.4. Gut Microbiota of Twins and Obesity

Turnbaugh et al. analyzed the composition of intestinal microbes in 154 monozygotic or dizygotic twins, concordant for leanness or obesity, and their mothers [68]. They aimed to assess the relationship between gut microbiota and host genotype and weight. The results proved that the composition of intestinal microbes is more similar between family members than unrelated individuals. Nevertheless, every subject presents with specific bacterial lineages, with a similar degree of co-variation between monozygotic and dizygotic twin pairs. Obesity was associated with phylum-level changes in microbiota: 16S pyrosequencing analysis of fecal samples revealed a lower proportion of Bacteroidetes and a higher proportion of Actinobacteria in obese compared with lean individuals. On the other hand, obese and lean subjects showed no differences in the proportion of Firmicutes. In recent years, studies on gut microbiota twins have sensibly increased according to the possibility of correlating gut microbiota to BMI and identifying a possible dependence on the host genotype. Lee et al. compared the composition of the fecal microbiota of Korean and US adult twins, in order to identify the influence of environmental and/or genetic factors on gut microbial composition [69]. The findings failed to reveal a statistically significant difference between the two populations. However, diversity was significantly lower in US obese twins that in lean twins. A similar trend that did not reach the level of statistical significance was noted in the smaller Korean sample. This study mostly revealed that interpersonal differences in fecal bacterial community structures were lower within a family than between families, and these are comparable for adult monozygotic and dizygotic twins.

However, these studies included exclusively twins with concordant body mass phenotypes. Another recent work characterized fecal microbiota of 20 Finnish monozygotic twin pairs employing qPCR and DGGE: 9 twin pairs were concordant and 11 discordant for BMI, and the participants were studied both individually and as twin pairs [58]. The study aimed to evaluate a possible correlation between diet and number or diversity of predominant fecal bacterial groups, in particular *Eubacterium rectale* group, *Clostridium leptum* group and *Bacteroides* spp., but also *Lactobacilli* and *Bifidobacteria*. The number of bacteria within the different bacterial groups did not differ between BMI groups. Moreover, looking at the possible association between nutritional intake and fecal microbiota, the authors found that individuals with high energy intake had significantly lower numbers of *Bacteroides* spp. (*p* = 0.007) and slightly high number of *Bifidobacteria* (*p* = 0.02) than individuals with lower energy intake. The greater mono-unsaturated fatty acids (MUFA) consumption was associated with lower bifidobacterial numbers (*p* = 0.0005); soluble fiber intake had a positive association with the *Bacteroides* spp. numbers (*p* = 0.009).

Furthermore, Tims et al. studied the fecal microbial composition in monozygotic twin pairs concordant and discordant in BMI to investigate if bacterial signatures correlate directly with BMI differences independent of the host genotype [61]. The human intestinal tract chip (*HITChip*), a phylogenetic microarray that has been benchmarked against several classical 16s rRNA gene-based methodologies, has been proven to be a valuable instrument in the study of fecal microbial diversity and composition. Both monozygotic twins, concordant and discordant for BMI, showed a significantly higher similarity of their microbiota profile compared with randomly paired subjects (*p* = 0.001). In addition, there was an inverse correlation between *Clostridium cluster IV* diversity and BMI and a positive correlation between *Eubacterium ventriosum/Roseburia intestinalis* and BMI. No consistent *Bacteroidetes/Firmicutes* ratios were observed in the pair-wise comparison of lower- and higher-BMI siblings. 

### 4.5. Gut Microbiota in Obese Children

An increase of body weight in children has been one of the most concerning public health issues of the present century. The prevalence of childhood obesity has risen at an alarming rate with WHO estimating that in 2010 the number of overweight children under the age of five years was over 42 million [1]. A prospective study has employed fluorescent in situ hybridization (FISH) to analyze the fecal microbiota composition of 25 obese/overweight and 24 normal children at 5 and 12 years of age, respectively, and compared with any change in BMI [43]. *Bifidobacteria* number during infancy was higher in children remaining at normal weight than in children becoming overweight (*p* = 0.02). On the other hand, reduced levels of *S. aureus* were linked to normal weight. The results indicated for the first time that differences in the gut microbiota may precede overweight development. Another work validated the finding of reduced levels of bifidobacteria in gut microbiota (*p* = 0.087) of children become obese at the age of 10 years vs. when they were three months old [70]. Early breastfeeding and diet seem to have also a determinant role in obesity development. Average concentrations of adiponectin in maternal colostrums resulted significantly higher in women with normal-weight children (at 10 years of age) than in mothers of overweight children. Very interestingly, the consumption of energy, carbohydrates, fat and proteins has been reported to not differ significantly different between obese and non-obese Indian children [48]. Furthermore, differences of dominant fecal microbiota in obese children compared with their normal peers were assessed to verify whether gut microbiota was a crucial actor in obesity development. Quantitative PCR studies showed no significant differences in the levels of *Bacteroides-Prevotella* group, *Eubacterium rectale*, *Bifidobacterium* group and *Lactobacillus acidophilus* group between obese and non-obese Indian children. Nevertheless, obese subjects had significantly higher levels of *Fecalibacterium prausnitzii*, a strain of *Firmicutes* able to ferment unabsorbed carbohydrates. Interestingly, presence of this bacterium in high concentrations in children can lead to increased energy harvesting from unabsorbed carbohydrates. Another Egyptian study also examined Bacteroidetes and Firmicutes frequencies in the stool of obese and normal-weight individuals, both children and adults, and found that obesity was associated with increased levels of both Firmicutes and Bacteroidetes (*p* = 0.03, *p* = 0.05, respectively) [52]. A comparison of the microbiota of lean and obese children was carried out by Payne et al. together with the evaluation of the fecal metabolite concentration [52]. Numerical variations in bacterial population numbers between obese and normal-weight children were not statistically significant for any population tested. They did not identify any correlation between Firmicutes/Bacteroides ratio and childhood obesity. These data are in disagreement with studies in adults. However, the analysis of fecal metabolites concentration revealed a significantly lower concentration of intermediate metabolic products in obese children, suggesting an exhaustive substrate utilization by obese gut microbiota. Thus, it was hypothesized that dysbiosis could be involved in the etiology of childhood obesity. Thus, the increased Firmicutes/Bacteroidetes ratio observed in obese adults could be a result of this dysbiosis. The same study group assessed also the impact of dietary energy on gut microbial communities and metabolism using a three-stage in vitro continuous fermentation model [71]. Two fecal samples from obese and lean children, respectively, were incubated with immobilized fecal microbiota—three different fermentation media were designed to examine the effects of prevalent Western diet on gut microbiota. A different composition of incubation medium was used in order to resemble the obese (high energy), normal weight (normal energy), and anorectic (low energy) child dietary intakes, and was alternately supplied to each microbiota during distinct fermentation periods. Gut microbial communities demonstrated differential metabolic and compositional adaptation response according to the different substrate provided: high energy medium was strongly butyrogenic, resulting in significant stimulation of butyrate-producing members of *Clostridia cluster IV*—normal and low energy nutrient loads induced significantly less metabolic activity, with a significant reduction in fermentable energy. This significant metabolic microbiota adaptation to nutrient load can be implied in microbial alterations observed in adults [71].

On the contrary a case-control study reported a negative correlation between Bacteroidetes and Bacteoridetes/Firmicutes ratio with BMI, suggesting that modifications in the major phyla of the intestinal microbiota could be the cause of obesity in Kazakh children [56]. Bervoets et al. carried out a genera-level research by quantitative culturing to identify the concentration of *Bacteroides fragilis*, *Bifidobacterium*, *Clostridium*, *Staphylococcus,* and *Lactobacillus* in lean and obese children [60]. Findings from this study are in agreement with those of a subsequent Spanish investigation, conducted through a comparative metagenomic investigation of gut microbial communities of obese and lean adolescents [59]. There was an elevated *Firmicutes/Bacteroidetes* ratio within the gut microbiota of obese children and adolescents. *Bacteroides fragilis* group and *Clostridium* spp. were borderline, but not significantly different between obese/overweight group and lean group (*p* = 0.05 and *p* = 0.074 respectively). On the other hand, fecal concentration of *Lactobacillus* spp. was significantly higher in the first group compared to lean children and adolescents (*p* = 0.035). The relevance of this study mainly consists in the association between energy intake and changes in gut microbiota composition. In fact, children and adolescents with higher energy intake possessed higher fecal concentrations of *Staphylococcus* spp. regardless of BMI status. On the opposite, Vael et al. in a prospective study demonstrated that high intestinal *Bacteroides fragilis* and low *Staphylococcus* concentrations in infants between three weeks and one year of age were associated with a higher risk of obesity later in life [53]. Finally, Nadal et al. evaluated the effects of obesity treatment programs on the fecal microbiota composition [46]. They found significantly reduced levels of *Clostridium hystoliticum*, *Eubacterium rectale* and *Clostridium coccoides* (part of *Firmicutes* phylum) correlating with weight loss in obese adolescents. Furthermore, Santacruz et al. found significantly lower *Clostridium* and *Bifidobacteria* levels in obese adolescents after an obesity treatment program [47].

### 4.6. Gut Microbiota and Obesity in Pregnant Women

Collado et al. noted significant microbial composition differences in pregnant women according to their BMI [42]: specifically, an overall higher number of bacteria was observed between the first and the third trimester in normal-weight and overweight women before pregnancy. However, significant differences were observed in microbial community composition among groups. Mainly, higher numbers of *Bacteroides* and *Staphylococcus aureus* were detected by FCM-FISH and qPCR in overweight compared vs. normal-weight women. Interestingly, microbiota composition also varied in relation to the amount of weight gain throughout pregnancy. *Bacteroides* showed a positive correlation with both weight and BMI before pregnancy (*p* = 0.005, *p* = 0.023) and, with weight gain over pregnancy (*p* = 0.014). In addition, every kilogram of weight gain was proportionally accompanied by 0.006 log units increase in the number of *Bacteroides*. On the other hand, *Bifidobacterium* number seemed to be higher in women who exhibited normal weight in pregnancy (*p* = 0.03). The concentration of *Clostridium* group during the first trimester of pregnancy showed a correlation with BMI (*p* = 0.067).

Santacruz et al. investigated the fecal microbiota of 50 pregnant women belonging to two groups, overweight or normal weight, based on their BMI [49]. Higher numbers of *Staphylococcus* and lower numbers of *Bifidobacterium* were detected in overweight women, perhaps in agreement with findings from the study by Collado [42]. Nevertheless, another study reported opposite findings: lower *Bacteroides* levels in overweight women. Increased numbers of *Enterobacteriaceae* and in particular of *E. coli*, were also associated with overweight women. Indeed, gut microbiota of women gaining excessive weight during pregnancy underwent similar changes as those of overweight women before pregnancy. Santacruz also investigated the relationship between gut microbiota composition change over pregnancy and biochemical parameters. Increased *Staphylococcus* concentration significantly correlated with increased serum levels of cholesterol. A raised number of Enterobacteriaceae and *E. coli* was linked with increased levels of serum ferritin and decreased levels of transferrin while a greater count of *Bifidobacteria* correlated with reduced levels of ferritin, saturation transferring index and increased levels of transferrin and folic acid. Finally, increased *Bacteroides* numbers were associated with higher levels of high-density lipoprotein (HDL)-cholesterol, folic acid and lower levels of triacylglycerol.

## 5. Mechanism Linking the Microbiota to Obesity

These shreds of evidence altogether have confirmed a statistical association between obesity and gut microbiota peculiar composition. A number of mechanisms has been proposed for gut microbiota causative action in obesity physiopathology. In fact, gut commensal bacteria interact with our metabolism at several points: it helps to convert ingested complex nutrients to SCFAs, transforms mucins and dietary fibers into simple sugars ready for absorption, stimulates intestinal epithelial proliferation, favors nutrients absorption and metabolism, it is the main actor in the shaping of gut crucial defense barrier constituted by systemic and mucosal immune system, and it activates bio-inactive compounds [72]. Nevertheless, gut microbiota plays an important role in human adipose tissue formation and deposition. Indeed, our intestinal bacteria are able to maintain the human body energy balance mainly because of their ability to share otherwise indigestible components of mammalians diet [73]. 

Germ-free (GF) mice have provided a complementary approach for characterizing the properties of human gut microbiota in the frame of metabolic processes. Backhed et al. have suggested that gut microbiota may regulate energy storage through host signaling pathways, analyzing GF and conventionalized mice [74]. Colonization of adult GF mice with a normal microbiota harvested from the cecum of conventionally raised animals produced a 57% increase in total body fat content and 61% increase of epididymal fat pads weight, despite reduced food intake. Moreover, the presence of microbiota increased serum levels of glucose and SCFAs that induced triglyceride production in the liver, increased adiposity, and reduced glucose tolerance. The authors observed also that the presence of gut microbes promoted an increased monosaccharide uptake from the gut, and an increased ability to degrade polysaccharides. 

Lemas et al. analyzed the relationship between human milk hormones, namely leptin and insulin, and both the taxonomic organization and metabolic properties of the infant microbiota [75]. The authors found increased levels of lipoprotein lipase (LPL) in epididymal fat pads of conventionalized mice. LPL, a key regulator of fatty acid release from triglyceride-rich lipoproteins, lead to the increased cellular uptake of fatty acids and adipocyte triglyceride accumulation. Another study consolidated evidence showing that gut microbiota is able to influence an important gut-derived host lipid metabolism factor, namely fasting-induced adipose factor (Fiaf), a circulating lipoprotein lipase inhibitor whose gene expression is normally selectively suppressed in the gut epithelium only by microbes although it is produced in liver and adipose tissue [76]. Indeed, GF mice showed increased gut expression of Fiaf, but administration of microbes from a normal mouse decreases such expression and leads to larger deposits of triglycerides in adipose tissue. 

### 5.1. SCFA and Energy Harvesting

Gut microbes are able to ferment starch, unabsorbed sugars, cellulosic and non-cellulosic polysaccharides, and mucins into SCFAs and gases in the intestine. The type and amount of SCFAs and gases produced in the gut depend on multiple factors including diet, (e.g., the availability of non-digested carbohydrates), gut microbial community composition, colonic transit time, and the segment of the colon where fatty acids are produced. Main SCFAs, produced as a result of carbohydrate and protein fermentation are acetate, propionate, and butyrate [77]. These SCFAs represent an additional source of energy deeply investigated by Schwiertz et al. [41]. Fecal samples of obese subjects had a 20% higher mean total SCFAs concentration than those from lean volunteers (*p* = 0.024). The degree of SCFAs increase was considerable, accounting for 41% of propionate, 28% of butyrate, vs. a moderate increase of valerate and acetate of 21% and 18%, respectively. Duncan et al. quantified changes in fecal SCFAs in response to changes in the dietary intake of carbohydrates [78]. They found that total SCFAs concentrations were lower during consumption of the high protein-low carbohydrate and high protein–moderate carbohydrate diets than during the maintenance period (*p* < 0.001). While the concentrations of the predominant SCFAs (acetate, propionate and valerate) significantly decreased due to the shift from the maintenance to the low carbohydrate diet (50%), butyrate levels decreased even more dramatically (75%). Moreover, the relationship between carbohydrate intake and butyrate concentrations was linear (r = 0.76, *p* < 0.001). Subsequently, Patil et al. confirmed a significant over-production of SCFAs in obese subjects through saccharolytic microbiota fermentation [54]. This evidence seemed to be associated with elevated archaeal density in the colon. On the other hand, Murphy et al. found that the fecal energy content of *ob/ob* mice was decreased and cecal SCFAs concentrations increased at seven weeks vs. lean controls [79]. These patterns were not stable over time as mostly fecal acetate levels decreased progressively. This study indicated also that SCFA levels were not related to modifications in proportions of Firmicutes, Bacteroidetes or Actinobacteria. Thus, the relationship between gut microbial composition and energy harvesting capacity of the body appears to be overall more complex than expected.

### 5.2. Gut Microbes and Inflammation

Low-grade metabolic inflammation constitutes a key contributor to obesity and metabolic syndrome and numerous works have proved a raise in pro-inflammatory cytokines in such conditions [80]. Lipopolysaccharide (LPS) endotoxin, an essential molecule of the cell walls of Gram-negative bacteria such as Bacteroidetes, stimulates adipose tissue deposition, increasing inflammation grade and insulin resistance [81]. Cani et al. firstly indicated that bacterial LPS plays a key trigger role in metabolic diseases linked to high-fat diet [82]. The results also demonstrated that high-fat feeding increased LPS levels over all day, as compared to controls. In addition, diurnal variations in plasma LPS concentration called “metabolic endotoxemia” were recorded. Subsequently, in order to causally link high-fat diet increased LPS concentrations to metabolic disease outbreak, authors measured LPS concentrations of high-fat feeding by continuously infusing LPS or saline in mice for a month. Fasted glycemia, fasted insulinemia, liver triglyceride content and body weight levels were greater in mice infused with LPS than those infused with saline. Furthermore, the entity of weight gain and visceral and subcutaneous adiposity in LPS infused mice were similar to those observed in mice fed with a high-fat diet. In addition, metabolic endotoxemia triggered the expression of inflammatory cytokines, similarly to high-fat diet. 

Other molecules induced by LPS are serum amyloid A (SAA) proteins, candidate mediators of inflammation and atherosclerosis. These proteins reach higher blood levels in obese patients [83]. Reigstad et al. found that the murine isoform of SAA3 levels in adipose tissue were significantly higher in conventionalized mice vs. GF mice [84]. They identified epithelial cells and macrophages as cellular sources of SAA3 in the colon and found that colonic epithelial expression of SAA3 may be part of an NF-kB-dependent response to LPS from gut bacteria. The study confirmed that LPS, and potentially other products of the indigenous gut microbiota, might elevate cytokine expression in tissues and exacerbate chronic low-grade inflammation observed in obesity.

### 5.3. Gut Microbes and Entero-Endocrine Cells

Energy intake and expenditure is also regulated through endocrine cells signaling from the intestine to the brain. Entero-endocrine cells in the gut respond to nutrient intake by secreting incretin hormones such as glucagon-like peptide 1 and 2 (GLP-1 and GLP-2) [85]. GLP-1 stimulates insulin release from the pancreas, slows gastric emptying, promotes satiety and weight loss, whereas GLP-2 enhances intestinal glucose transport and reduces intestinal permeability. Gut microbiota is able to regulate entero-endocrine cells functioning [86]. Obesity-associated intestinal microbiota produces more SCFAs from carbohydrate fermentation than lean controls, and entero-endocrine cells express a G-protein coupled 41 (Grp41), a receptor for SCFAs, that can be found also in small intestinal, colonic and adipocyte epithelium; it is necessary for the metabolic effects of these microbial metabolites. Interestingly, mice lacking Grp41 have reduced levels of the gut hormone PYY, delayed gut transit time, lower intestinal absorption of SCFAs from the diet, and lower fat accumulation in fat pads: all these effects are related to a decreased energy harvesting from the diet [87].

## 6. Probiotics and Obesity

### 6.1. Main Probiotics Used in Obesity Treatment in Animals and Humans

As emerged from the cited literature, it has been proven that several probiotics, used alone or in symbiotic mixtures, are able to exert their anti-obesity effects through species- and strain-specific mechanisms (e.g., gut microbiota modulation, lower insulin resistance, greater satiety). More specifcally, *Lactobacillus* (e.g., *L. Casei* strain *Shirota* (LAB13), *L. Gasseri*, *L. Rhamnosus*, *L. Plantarum*) and *Bifidobacterium* (e.g., *B. Infantis*, *B. Longum*, and *B. Breve B3*) species have been successfully used in well-established animal models of obesity due to their low pathogenicity and low level of antibiotic resistance [88]. These treatments led to different degrees of less weight gain and reduced fat accumulation vs. placebo arms. However, these findings have not been confirmed in other trials [89]. 

Studies conducted on human subjects can be distinguished in pediatric and adolescent vs. adult studies. 

*Lactobacillus Rhamnosus* GG supplementation one month before the expected delivery and child treatment for further six months lower weight gain in this subset of subjects (age range 1–4 years). However, this result was not present 10 years later [89].

Interestingly, the use of supplementation based on *L. Salivalis* ls-33 or VSL#33 [90] in obese adolescents failed to reduce body weight, waist circumference, and visceral fat (Table 3).

On the other hand, VSL#3 (a mixture of *Streptococcus Thermophilus* DSM24731, *L. Acidophilus* DSM24735, *L. Delbrueckii* subsp. *Bulgaricus* DSM24724, *L. Paracasei* DSM24733, *L. Plantarum* DSM24730, *B. Longum* DSM24736, *B. Infantis* DSM24737, and *B. Breve* DSM24732) and another probiotics mixture (namely, *L. Acidophilus* ATCC B3208, *L. Rhamnosus* DSMZ 21690, *B. Lactis* DSMZ 32,296, and *B. Bifidum* ATCC SD6576) showed beneficial effects on BMI, fatty liver, insulin resistance, and GLP-1 levels in treated children [91,92]. Furthermore, body weight was significantly reduced after *B. Pseudocatenulatum* CECT 7765 administration in obese children with insulin resistance [93]. 

Other studies failed to confirm these positive results (e.g., trials with *L. Salivalis* ls-33, VSL#33) [94,95] (Table 3). 

Discrepancies between findings from different studies may be explained by different experimental setup involving sometimes effective life-style changes and sometimes not. 

In adults, different strains of *Lactobacillus* and *Bifidobacterium*, alone or in combination, and *Pediococcus Pentosaceus*, led to a significant reduction of body weight, BMI, waist circumference, and fat mass [96,97,98,99,100,101] (Table 3).

As administered dosage of probiotics affects efficacy of treatment, reduced visceral adiposity and waist circumference were observed after exposure to a high dose of *L. Gasseri* BNR17 [99]. These results were not so univoque after different doses of Ecologic^®^ (a mixture of multi-strains of *Lactobacillus* and *Bifidobacterium*), although this was a study conducted in obese women only [102].

Interestingly, only a report showed by Sánchez et al. showed gender-specific effects of probiotics in human obese subjects. Indeed, *L. Rhamnosus* CGMCC1.3724 and a restricted caloric diet administration showed a significantly higher weight loss in obese women vs. men. This finding can be explained by a greater impact on satiety feeling, eating habits, and mood in women vs. men [103]. 

Finally, it is important to note that there are few evidences on the potential preventive effect on obesity of some probiotics in non-obese subjects. In detail, VSL#3 is able to reduce body weight and fat accumulation such as *L. Gasseri* SBT2055 administration [104,105] (Table 3).

### 6.2. Mechanisms of Action of Probiotics Efficacy in Obesity

According to the actions exerted by human gut microbiota in humans, widely described above, we can recognize three main mechanisms of action in obesity treatment by probiotics: antagonistic effects on pathogenic microorganism growth and competitive adherence to intestinal mucosa and epithelium (antimicrobial activity), increased intestinal mucus layer production and reduced intestinal permeability (barrier function), and modulation of the gastrointestinal immune system (immunomodulation) [106]. Altogether, these mechanisms can modulate gut microbiota composition and host metabolism, restoring a “lean gut microbiota” [88,107,108].

## 7. Conclusions and Future Perspectives

The debate on the significance of the correlation between gut microbiota imbalance and obesity is one of the hottest topics in medicine. Although several molecular pathways have widened the view on the causative association between gut microbiota alterations and obesity development, this linkage remains very complex. On the other hand, the obesity pandemic asks for a solid response able to restore the significant gut microbial imbalance present in these patients. Thus, these findings imply the possibility and need for therapeutic manipulation of intestinal microbiota to prevent or treat obesity and its metabolic manifestations.

The correlation between Firmicutes/Bacteroidetes ratio and obesity constitutes strong evidence arising from 30 years of research in this filed. However, several recent studies have highlighted the complexity of the altered composition of intestinal microbiota in obese patients compared with lean subjects. Therefore, each study has linked obesity to species- or genus-specific composition profiles. The extreme variability of the results can be attributed to the different experimental designs, microbiota fingerprinting, and genome analyses. We must also mention the different populations or sub-populations studied. 

Particularly, the heterogeneity of methods used to quantify the levels of gut microbiota does not allow a proper comparison of the results generated by different studies, as every technique is biased by accuracy, sensitivity or specificity issues. Thus, there is the need for a standardization of techniques to be used to detect and classify gut microbiota composition in obese subjects. 

In more recent years, the attention of researchers has focused on the understanding of the specific metabolic patterns linked to the obesity physiopathology. Intestinal bacteria are an important part of these integrated functional networks. It has derived an increasing interest of investigators for the impact of gut microbiota modulation by the diet in these metabolic processes. 

In conclusion, further investigations using standardized next-generation sequencing technologies should be conducted on the real association of gut microbiota composition and specific obesity-related phenotypes. Moreover, the complex interaction of intestinal bacteria with the host has to be unraveled, as well as the possible effect of variables such as diet, age, gender or physical activity. Future evidence can help, using the modulation of these variables in order to re-shape gut microbiota in a healthier profile. Indeed, it remains possible to directly modulate gut microbiota with probiotics, prebiotics, antibiotics, or other therapeutic interventions. Although several RCTs on probiotics in obesity have been carried out, their results are not yet convincing. Thus, more randomized placebo-controlled are lacking in this respect. 

## Figures and Tables

**Table 1:** Main bacteria and Archaea in the human gut microbiota.

Domain	Phylum	Class	Order	Family	Genus
*Bacteria*	*Bacteroidetes*	Bacteroidia	Bacteroidales	Bacteroidacee	Bacteroides
				Prevotellacee	Prevotella
					Xylanibacter
				Rikenellacee	Rikenella
	*Firmicutes*	Clostridia	Clostridiales	Clostridiacee	Clostridium
				Ruminococcae	Faecalibacterium
					Ruminococcus
				Peptostreptococcae	Peptostreptococcus
					Fusibacter
				Eubacteriacee	Eubacterium
				Veillonellacee	Veillonella
				Lachnospiraceae	Roseburia
		Bacilli	Bacillales	Bacillaceae	Bacillus
				Lysteriaceae	Lysteria
				Staphylococcaceae	Staphylococcus
				Pasteuriaceae	Pasteuria
			Lactobacillales	Lactobacillaceae	Lactobacillus
				Enterococcaceae	Enterococcus
				Streptococcaceae	Streptococcus
	*Actinobacteria*	Actinobacteria	Bifidobacteriales	Bifidobacteriaceae	Bifidobacterium
					Gardnerella
			Actinomycetales	Actinomycetaceae	Actynomices
	*Proteobacteria*	Deltaproteobacteria	Desulfobacteriales	Desulfobulbaceae	Desulfovibrio
		Gammaproteobacteria	Enterobacteriales	Enterobacteriaceae	Escherichia
					Enterobacter
					Klebsiella
					Proteus
		Epsilonproteobacteria	Campylobacteriales	Campylobacteriaceae	Campylobacter
				Helycobacteriaceae	Helycobacter
	*Fusobacteria*	Fusobacteria	Fusobacteriales	Fusobacteriaceae	Fusobacterium
	*Verrucomicrobia*	Verrucomicrobiae	Verrucomicrobiales	Verrucomicrobiaceae	Verrucomicrobium
	*Synergistetes*	Synergistia	Synergistales	Synergistaceae	Synergistes
	*Spirochaetes*	Spirochaetes	Spirochaetales	Spirochaetaceae	Spirochaeta
					Treponema
	*Cyanobacteria*	Cyanobacteria			
*Archaea*	*Euryarchaeota*	Methanobacteria	Methanobacteriales	Methanobacteriaceae	Methanobrevibacter
					Methanobacterium
					Methanosphaera

**Table 2:** Gut microbial population and obesity: relationship, causality and effects in human studies.

Source	Study Subjects	Comparison	No. of Subjects	Methods	Community Measured	Major Findings
Ley et al. [32]	Human adults	Obese vs. controls	12 obese, 2 normal weight	16S rRNA sequencing	Bacteroidetes Firmicutes	Significantly reduced level of Bacteroidetes in obese subjects.
Collado et al. [42]	Pregnant women	Obese vs. lean pregnant	18 overweight, 36 normal weight pregnant women	FCM-FISH qPCR	*Bacteroides Bifidobacteria Staphylococcus aureus*	High numbers of *Bacteroides* group and *S.aureus* in the overweight pregnant women.
Zhang et al. [40]	Human adults	Obese vs. control vs. after RYGB	3 normal weight, 3 obese, 3 post-gastric bypass	16S Pyrosequencing qPCR	Firmicutes Bacteroidetes Proteobacteria Actinobacteria Fusobacteria Verrucomicrobia	More Bacteroidetes in obese subjects (not significant). *Prevotellacee* (phylum *Bacteroidetes*) and *Coriobacteriacee* (phylum *Actinobacteria*) increased in obese. Significant increase in *Methanobacteriales* in obese subjects.
Kalliomaki et al. [43]	Human children	Overweight/obese Normal weight	25 overweight: 7 obese, 24 normal weight	FISH	*Bifidobacteria Lactobacilli Clostridia Staphylococcus aureus*	Lower number of bifidobacteria and greaternumber of *S. aureus* predict Obese/overweight phenotype.
Duncan et al. [44]	Human male	Obese vs. normal weight	15 obese, 14 lean	FISH	Bacteroides Firmicutes *E.rectale/C. coccoides*	No differences in *Bacteroides* level in obese and normal weight subjects. Significant diet-dependent reduction in *Eubacterium rectale/C. coccoides* (phylum *Firmicutes*) levels in obese subjects.
Turnbaugh et al. [33]	Human twins	Obese and normal twins, mothers	154 subjects: 31 monozygotic twin pairs, 23 dizygotic twin pairs, 46 mothers	16S pyrosequencing V2 and V6 variable region	Bacteroidetes Firmicutes Proteobacteria Actinobacteria	Significantly reduced levels of Bacteroidetes in obese and increased level of Actinobacteria. Nearly half of the lean-enriched genes are from Bacteroidetes.
Armougom et al. [37]	Human adults	Anorexic, normal weight and obese	20 normal weight, 20 obese, 9 anorexic	qPCR	*Lactobacillus M. smithii* Bacteroidetes Firmicutes	Significantly reduced levels of Bacteroidetes in obese subjects versus healthy subjects (*p* < 0.01). Firmicutes data are similar in the three categories. Significantly higher levels of *Lactobacillus.* Increase of *M. smithii* in anorexic subjects (*p* < 0.05).
Mai et al. [45]	Human adults	African American and Caucasian American	98 subjects	FISH qPCR	Bacteroidetes *Clostridia cluster XIV* (Firmicutes)	No significant difference in *Bacteroidetes* numbers between obese and normal-weight subjects.
Nadal et al. [46]	Human adolescents	Before and after 10 weeks of calorie-restricted diet	39 overweight adolescents	FISH	Bacteroidetes*/Prevotella Bifidobacterium C. histolyticum E. rectale/C. coccoides Lactobacillus/En-terococcus*	Greater weight loss after a multidisciplinary treatment program associated with: significant reduction of *Eubacterium rectale, Clostridium coccoides* and *C. histolyticum*; significant increase in *Bacteroides/Prevotella*.
Santacruz et al. [47]	Human adolescents	Before and after diet and exercise for 10 weeks	36 obese adolescents	qPCR	*Bacteroides fragilis Lactobacillus C. coccoides C. leptum Bifidobacterium Escherichia coli*	After an obese group submitted to a weight program lost >4 Kg: significant reduction in *C.coccoides*; increase in the *Bacteroides fragilis* and *Lactobacillus* group.
Schwiertz et al. [41]	Human adults	Obese vs. overweight vs.normal weight	98 subjects: 30 lean, 35 overweight, 33 obese subjects	qPCR	Firmicutes Bacteroidetes *Bifidobacteria*	Significantly increased level of Bacteroidetes in obese subjects and decreased level of Firmicutes. Significant decrease in *Bifidobacteria* and *Methanobrevibacter* spp. in obese subjects.
Balamurugan et al. [48]	Human children	Obese vs. non obese	15 obese, 13 normal weight	qPCR	Bacteroidetes *Bifidobacterium Lactobacillus acidophilus E. rectale F. prausntzi*	No significant difference in *Bacteroides/Prevotella* and *Bifidobacterium* spp. Significant increase of *Fecalibacterium prausntzi* levels (*Firmicutes* species) in obese subjects.
Santacruz et al. [49]	Pregnant women	Overweight/obese pregnant women vs. normal weight women	16 overweight pregnant, 34 normal weight pregnant women	qPCR	*Bifidobacterium Lactobacilli Bacteroidetes Escherichia coli Staphilococcus*	Significant reduction of *Bifidobacterium* and *Bacteroides* numbers in obese pregnant women. Increased levels of *Staphilococcus* and *E. coli* in overweight women.
Abdallah Ismail et al. [50]	Human children and adults	Obese vs. normal weight	79 subjects: 51 obese, 28 normal weight	qPCR	Bacteroidetes Firmicutes	Significantly increased distribution of Bacteroidetes and Firmicutes in the obese group.
Furet et al. [51]	Obese after RYGB	Obese subjects enrolled in a bariatric-surgery program	30 obese after RYGB, 13 lean	qPCR	*Bacteroides/Prevotella E. Coli F. Prausnitzii Bifidobacterium Lactobacilli*	*Bacteroides*/*Prevotella* group was lower in obese subjects than in control subjects and increased after 3 months. *Escherichia coli* species after 3 months and inversely correlated with fat mass and leptin levels. *F. prausnitzii* species was lower in subjects with diabetes and associated negatively with inflammatory markers.
Zuo et al. [39]	Human adults	Obese vs. normal weight	52 obese, 52 normal weight	Culture	*Bacteroides Clostridium perfringens*	Significantly reduced levels of *Clostridium perfringens* and *Bacteroides* in obese population.
Payne et al. [52]	Human children	Obese vs. normal weight children	30 subjects: 15 obese, 15 normal weight	qPCR TGGE	Bacteroides Firmicutes *Roseburia/E.rectale Lactobacillus Bifidobacterium Enterobacteriacee F. prausnitzii*	No significant differences for any population tested between obese and normal weight children.
Vael et al. [53]	Human children	Children at 3, 26 and 52 weeks of age	138 subjects	Culture	*Bacteroides fragilis Bifidobacterium Lactobacillus Enterobacteriacea Staphylococcus Clostridium*	High intestinal *Bacteroides fragilis* and low *Staphylococcus* concentrations in infants between the age of 3 weeks and 1 year are associated with a higher risk of obesity later in life.
Patil et al. [54]	Human adults	Lean, normal, obese and surgically-treated obese subjects	20 subjects: 5 lean, 5 normal, 5 obese, 5 surgically treated	qPCR	*Bacteroidetes Firmicutes*	*Bacteroides* are prominent among the obese subjects.
Zupancic et al. [55]	Human adults	Stratified by BMI	310 adult subjects	16S rRNA pyrosequencing V1-V3	*Bacteroidetes* spp. *Firmicutes* spp.	Bacteroidetes/ Firmicutes ratio is not associated with BMI or metabolic syndrome traits.
Xu et al. [56]	Human children	Normal, overweight and obese subjects	175 children: 91 normal, 62 overweight, 22 obese	qPCR	*Bacteroidetes Firmicutes*	Reduction of Bacteroidetes level in obese group (*p* = 0.002).No differences in Firmicutes level between lean and obese children (*p* = 0.628).
Munukka et al. [57]	Premenopausal women	Overweight/obese women with and without metabolic disorders	85 premenopausal women	FISH	*Bacteroidetes Bifidobacterium* spp. *Enterobacteriacee E. rectale/C. coccoides F. prausnitzii*	Proportion of *E. rectale/C. coccoides* is higher in MDG women compared to NMDG and NWG women. Certain members of *E. rectale/C. coccoides* are associated with obesity related metabolic disease, not obesity per se.
Million et al. [38]	Human adults	Obese vs. normal weight	115 subjects: 68 obese, 47 controls	Culture (*Lactobacillus* spp.) qPCR	*Lactobacillus* spp. *Bacteroidetes Firmicutes M. smithii*	*L. paracasei* is significantly associated with lean status. *L. reuteri, L. gasseri* are significantly associated with obesity. *M. smithii* is less abundant in human obesity. Bacteroidetes are lower in obeses (not significant, *p* = 0.25)
Simões et al. [58]	Human twins	Obese, overweight, normal weight	20 twin pairs	qPCR DGGE	*Eubacterium rectale group Clostridium leptum group Lactobacillus group Bacteroides* spp.	The abundance and diversity of the bacterial groups not differ between normal weight, overweight and obese individuals. Diet plays an important role in the modulation of the stool microbiota, in particular *Bacteroides* spp. and *Bifidobacteria*
Ferrer et al. [59]	Human adolescents	Lean and obese subjects	1 obese, 1 lean individual	qPCR	Bacteroidetes Firmicutes Actinobacteria Proteobacteria	Lower Bacteroidetes abundance and greater frequencies of Clostridia (*Firmicutes* spp.) in obese subjects.
Million et al. [41]	Humans adults	Obese, overweight, lean and anorexic subjects	263 individuals: 134 obese, 38 overweight, 76 lean, 15 anorexic	qPCR	Bacteroidetes Firmicutes, *M. smithii Lactobacillus* spp. *E.coli*	*L. reuterii* was positively correlated with BMI. *M. smithii* was negatively associated with BMI. Bacteroidetes was not correlated with BMI.
Bervoets et al. [60]	Human children	Obese, overweight and morbidly obese (O/O group) and normal-weight, thinness (C group)	26 overweight/obese, 27 lean	qPCR Mass spectrometry	*Bacteroides Bifidobacterium Clostridium Staphylococcus Lactobacillus*	Higher concentration of *Lactobacillus* spp. in obese microbiota. Increased concentration of Firmicutes and decreased concentration of Bacteroidetes in obese children.
Tims et al. [61]	Human twins	Concordant and discordant BMI twin pairs	40 subjects: 20 discordant BMI 20 concordant BMI twin pairs	HITChip phylogenetic microarrays	Bacteroidetes Firmicutes Actinobacteria at phylotype level	MZ twins have more similar GI microbiota compared with unrelated subject. Inverse correlation between *Clostridium cluster IV* diversity and BMI; positive correllation between *Eubacterium ventriosum/Roseburia intestinalis* and BMI. No consistent Bacteroidetes/Firmicutes ratio were observed in pair-wise comparison of lower- and higher-BMI siblings.

**Table 3:** Probiotics and obesity: gut microbiota modulation in human studies.

Source	Study Subjects	Type of Trial	Probiotic(s) Administered	Placebo Arm	Duration	Major Findings
Gomes et al. [97]	43 obese Women (20–59 years); 21 probiotic/22 placebo	Randomized, double-blind, placebo-controlled, two arm, parallel-group	*L. acidophilus LA-14, L. casei LC-11, Lactococcuslactis LL-23, B. bifidum BB-06, B. lactis BL-4 (2* × *1010 CFU/day) + dietary intervention*	1 cap/day placebo + dietary prescription	8 weeks	Lower WC
Higashikawa et al. (2016) [98]	Overweight adults aged 20–70 years (*n* = 62); Intervention I (*n* = 21); Intervention II (*n* = 21); placebo (*n* = 20)	Randomized, double-blind, placebo-controlled clinical trial	*Intervention I: Living LP28*; *Intervention II Heat-killed LP28 (Pediococcus pentosaceus) (1011 CFU/day)*	1 cap/day placebo	12 weeks	<BMI and WC after Intervention II
Jung et al. (2015) [96]	Obese adults aged 20–65 years (*n* = 120); intervention (*n* = 60); placebo (*n* = 60)	Double-blind, placebo-controlled, randomized clinical trial	L. *curvatus* HY7601 + L. *plantarum* KY1032 (2.5 × 109 CFU of probiotics/2 cap/day) + healthy lifestyle habits	2 cap/day placebo + healthy lifestyle measures	12 weeks	<Body weight, WC and body fat
Kadooka et al. (2010) [104]	Adults aged 33-63 years with obese tendencies (*n* = 87); intervention (*n* = 43); control group (*n* = 44)	Multicenter, double-blind, randomized, placebo-controlled intervention trial	Fermented milk containing Lactobacillus gasseri SBT2055 (5 × 1010 CFU/100 g fermented milk). Intake of 200 g/day	200 g/day of fermented milk without probiotic	12 weeks	Lower abdominal, subcutaneous fat deposition, body weight and BMI
Kim et al. (2018) [99]	Obese adults aged 20–75 years (*n* = 90); low-dose intervention (*n* = 30); high-dose intervention (*n* = 30); placebo (*n* = 30)	Randomized, double-blind, placebo-controlled trial	Low (109 CFU/day) and high (1010 CFU/2 cap/twice a day) dose of Lactobacillus gasseri BNR17 + lifestyle changes	2 cap/twice a day of placebo + lifestyle changes	12 weeks	Lower visceral adipose tissue; lower WC in high-dose and low-dose groups
Luoto et al. (2010) [89]	Mother–child pairs (*n* = 113); intervention (*n* = 54); placebo (*n* = 59)	Randomized, double-blind, prospective follow-up study	Lactobacillus rhamnosus GG (1 × 1010 CFU/day)	1 cap/day of placebo (microcrystalline cellulose)	Mothers 4 weeks before expected delivery; in infants up to 6 month old	Lower weight gain at 1 year of life and 4 years; no changes in later stages of development
Minami et al. (2018) [100]	Healthy pre-obese adults aged 20–64 years (*n* = 80); intervention (*n* = 40); placebo (*n* = 40)	Randomized, double-blind, placebo-controlled trial	Bifidobacterium breve B-3 (1010 CFU/2 cap/day)	2 cap/day of placebo	12 weeks	<Body fat mass
Osterberg et al. (2015) [105]	Healthy non-obese young male adults (18–30 years) (*n* = 20); intervention (*n* = 9); placebo (*n* = 11)	Randomized, double-blind placebo-controlled clinical trial	Two vials of VSL#3 (450 billion bacteria per vial in milk shake/once a day) + high fat diet (HFD)	Two vials of placebo in milk shake/once a day + HFD	4 weeks	Lower weight and fat
Pedret et al. (2018) [101]	Abdominally obese adults (*n* = 126); Intervention I (*n* = 42); Intervention II (*n* = 44); placebo (*n* = 40)	Randomized, parallel, double-blind, placebo-controlled trial	Bifidobacterium animalis subsp. Lactis CECT 8145 (Intervention I) or its heat-killed form (Intervention II) (1010 CFU/cap/day)	1 cap/day of placebo	12 weeks	Lower BMI, WC and waist circumference/height ratio
Sánchez et al. (2017) [103]	Obese adults aged 18–55 years (*n* = 125); intervention (*n* = 62); placebo (*n* = 63)	Double-blind, randomized, placebo-controlled trial	L. rhamnosus CGMCC1.3724(1.62 × 108 CFU/2 cap/day) + healthy eating behavior	250 mg of maltodextrin + 3 mg magnesium stearate + eating advices	12 weeks	Lower weight
Sanchis-Chordá et al. (2018) [93]	Obese children (aged 10–15 years) with insulin resistance (n = 48); intervention (*n* = 23); placebo (*n* = 25)	Double-blind, randomized, placebo-controlled trial	B. pseudocatenulatum CECT 7765 (109 × 10 CFU/day) + dietary recommendations	Placebo + dietary recommendations	13 weeks	Lower weight
Szulinska et al. (2018) [102]	Obese postmenopausal women aged 45–70 years (*n* = 81); low-dose intervention (*n* = 27); high-dose intervention (*n* = 27); placebo (*n* = 27)	Randomized-double-blind, placebo-controlled clinical trial	Low (2.5 × 109 CFU/day) and high dose (1010 CFU/day/two sachets per day) of probiotic mixture including nine different strains of Lactobacillus and Bifidobacterium	1 cap/day of placebo	12 weeks	Lower body weight, BMI and fat mass in low and high-dose group; improved lipid profile in the high-dose group
Vajro et al. (2011) [90]	Obese children (aged 10–13 years) with hypertransaminasemia and ultrasonographic bright liver (*n* = 20); intervention (*n* = 10); placebo (*n* = 10)	Double-blind, placebo-controlled pilot study	Lactobacillus rhamnosus GG (12 billion CFU/day)	1 cap/day of placebo	8 weeks	Lower hypertransaminasemia; lower BMI and visceral fat adding lifestyle interventions

BMI: body mass index; WC: waist circumference.
